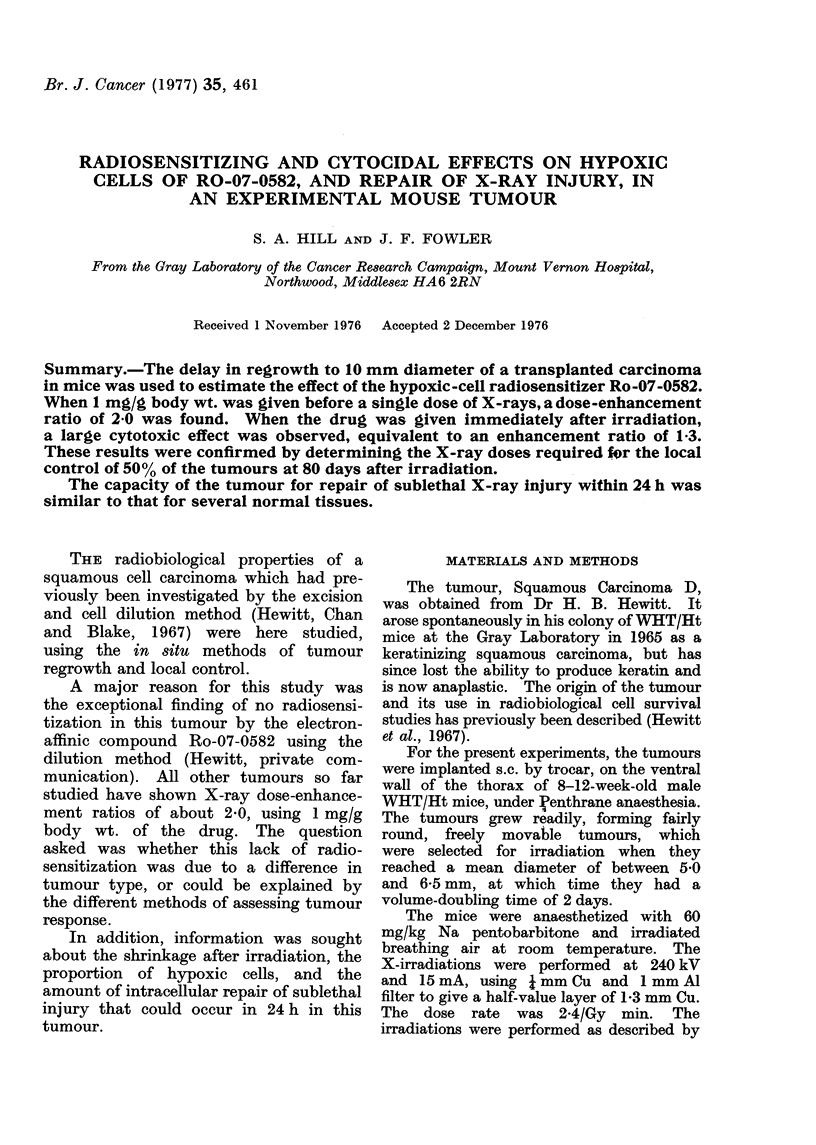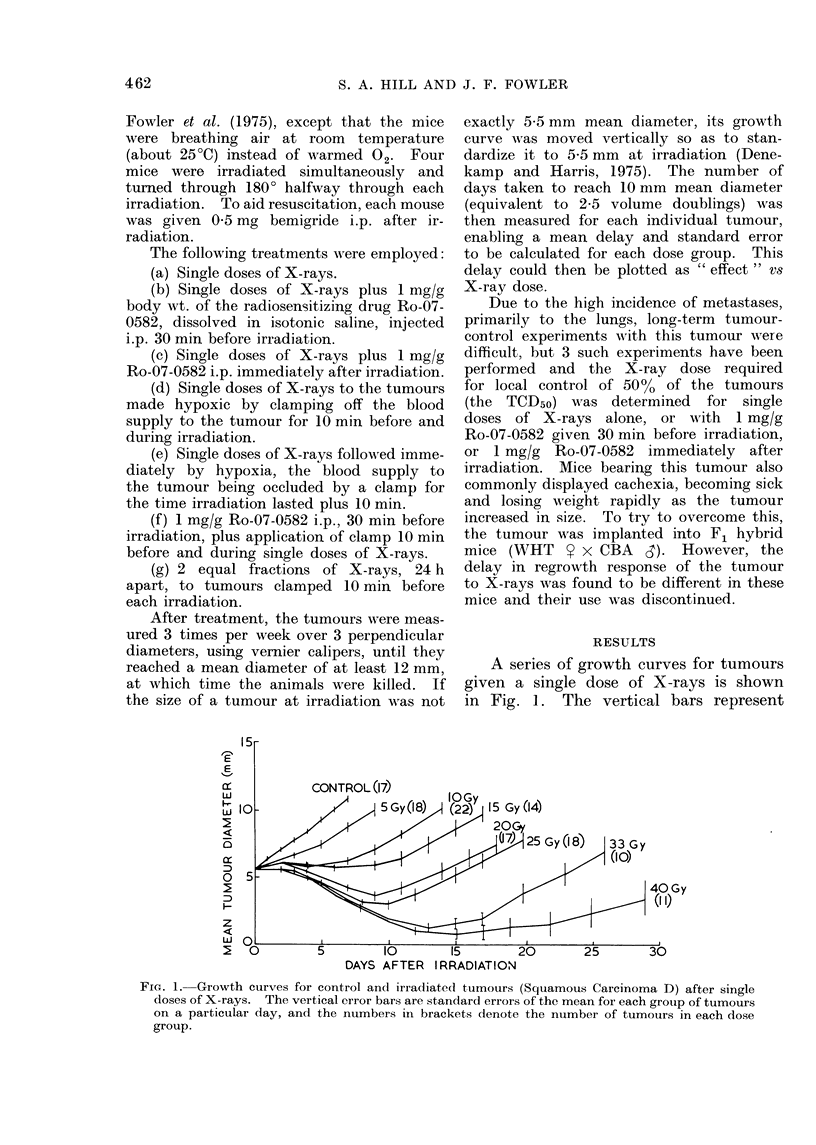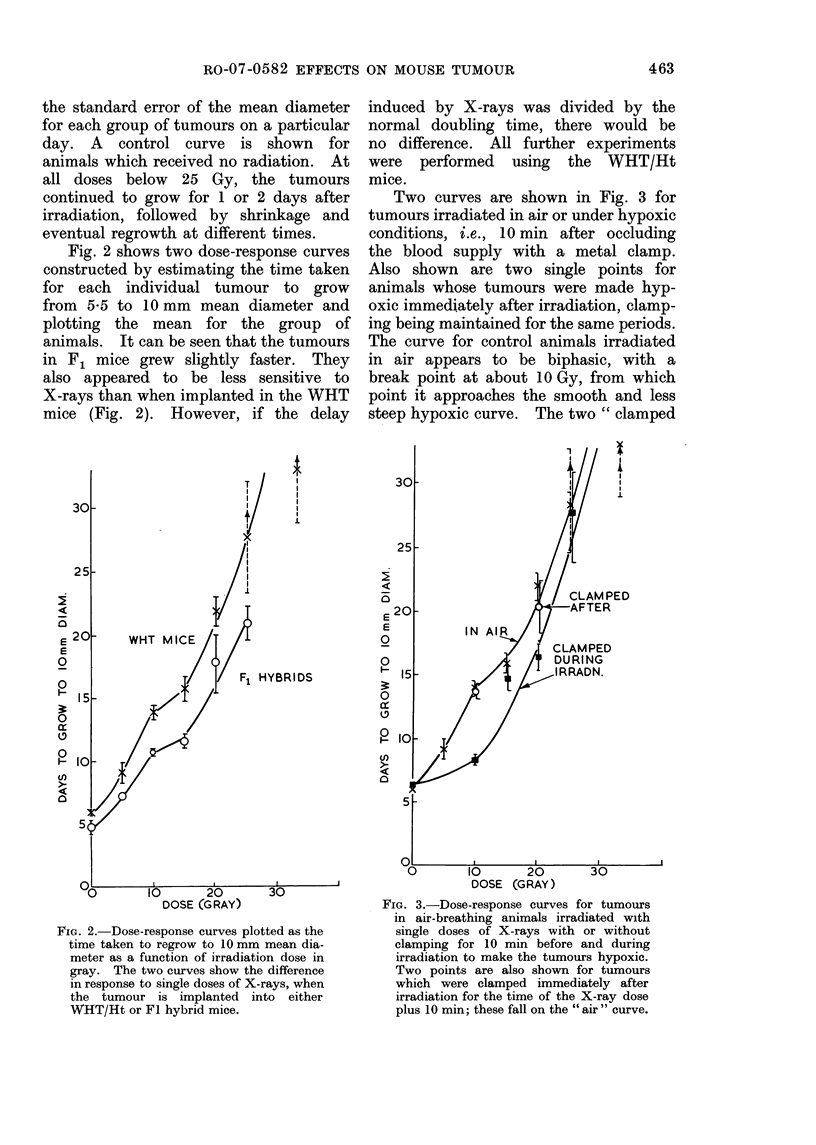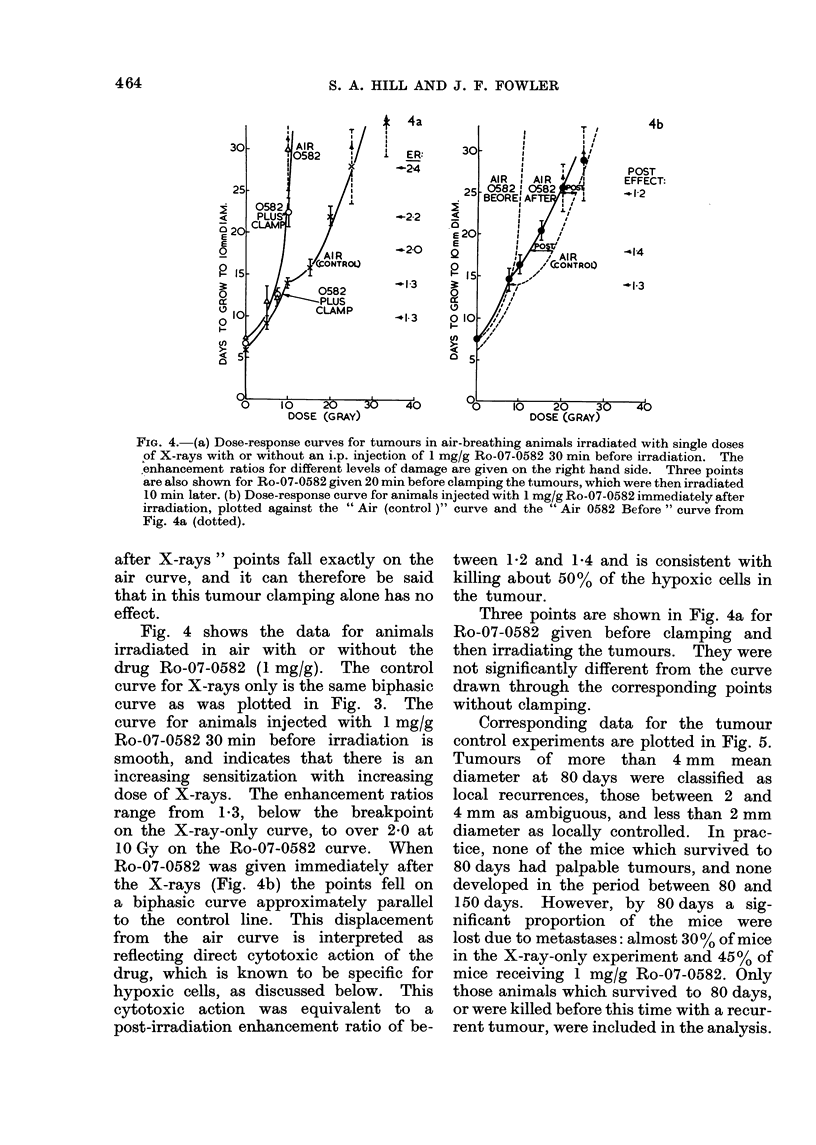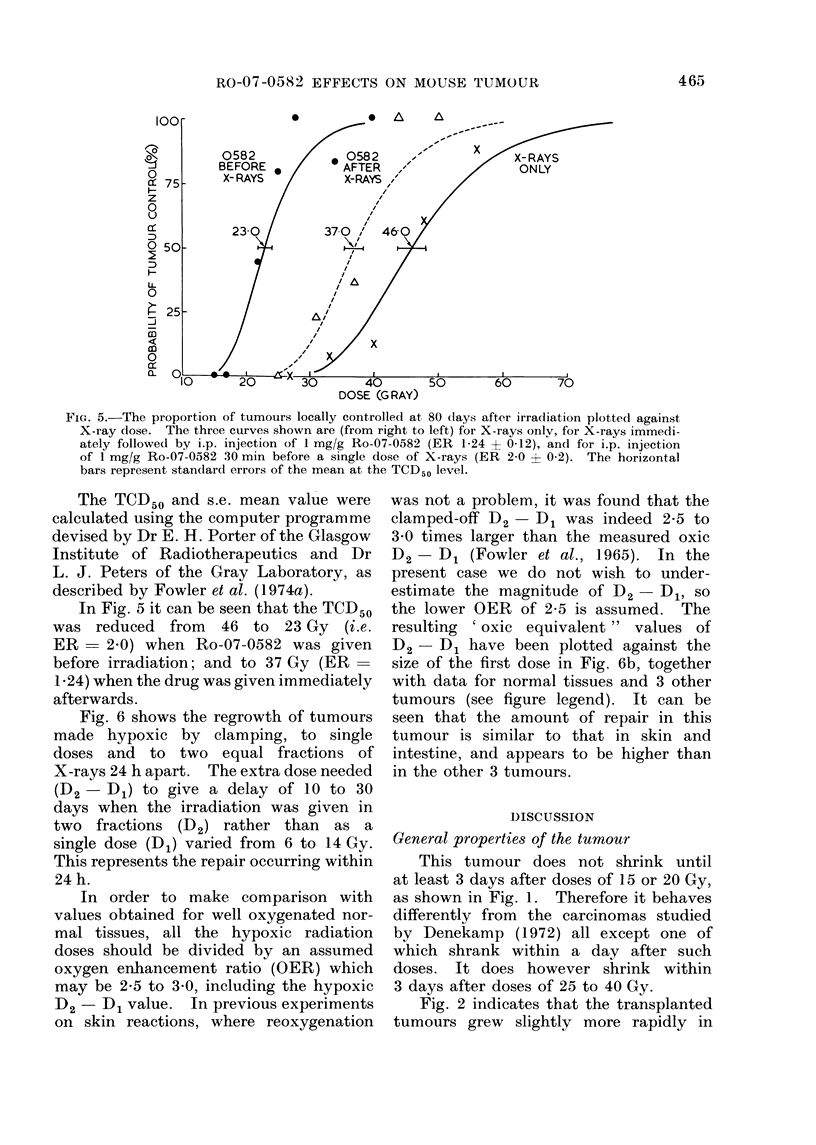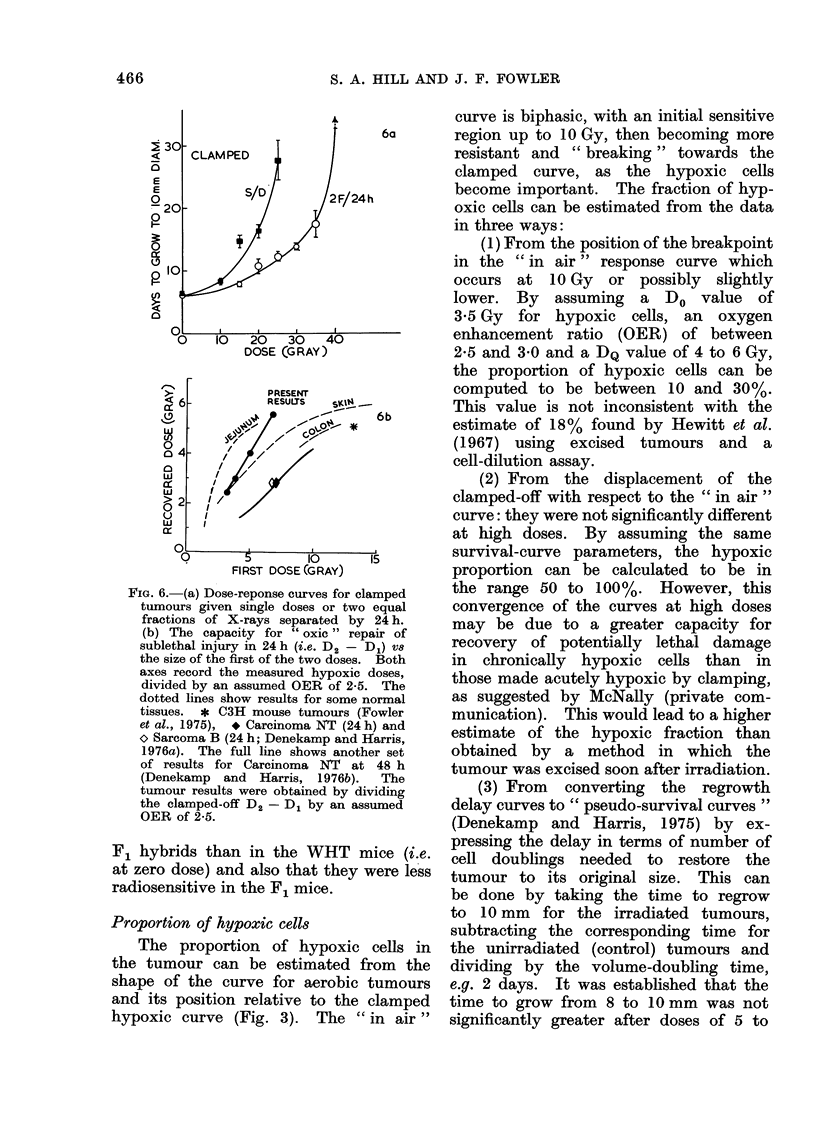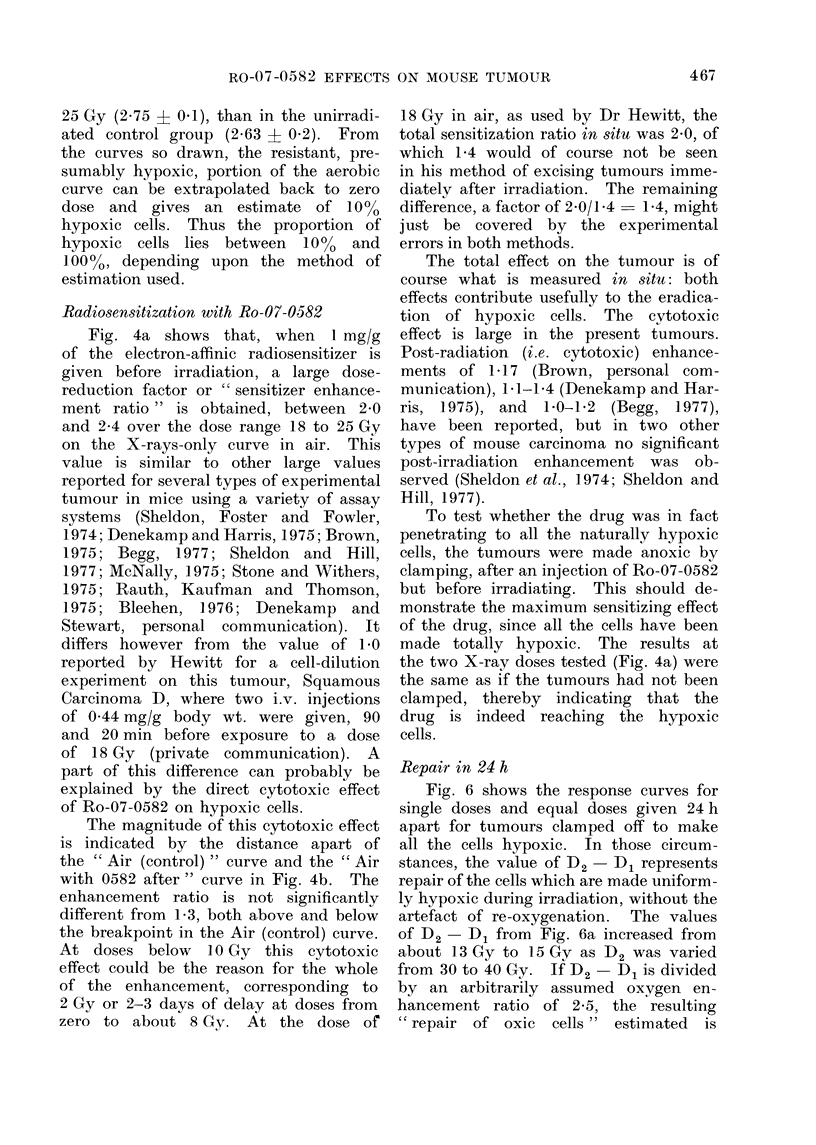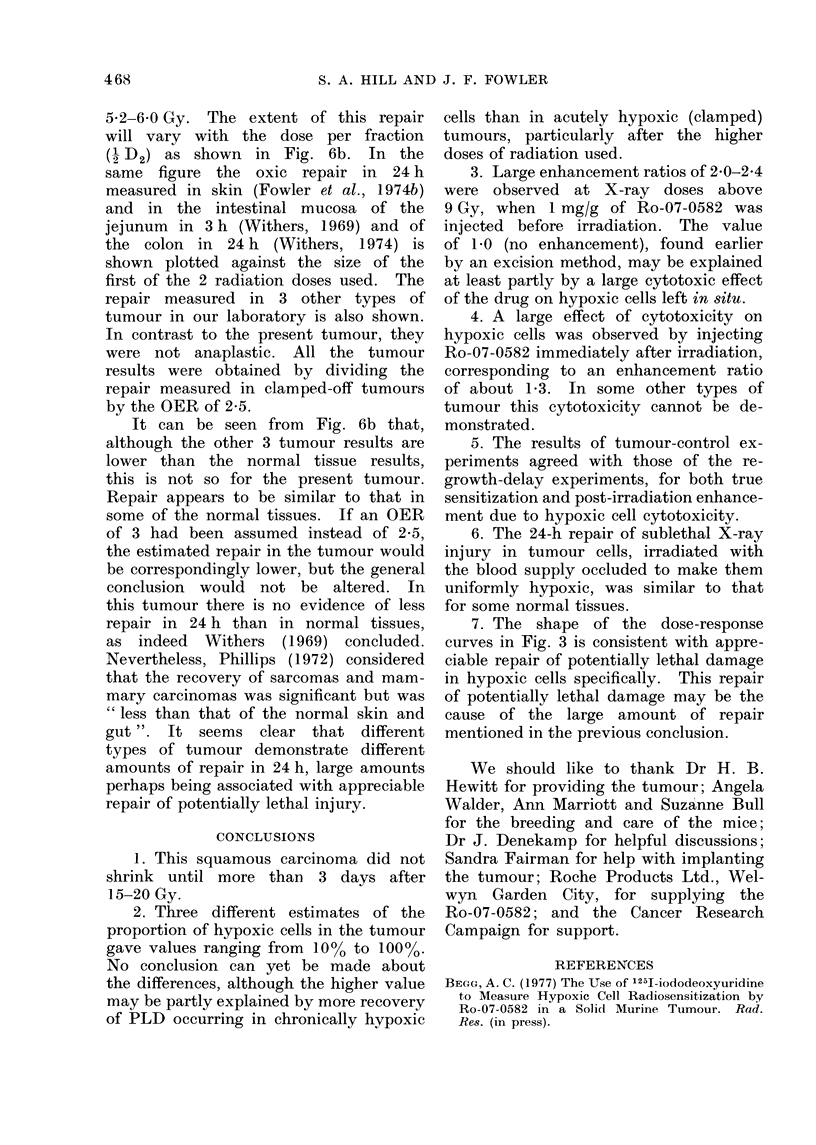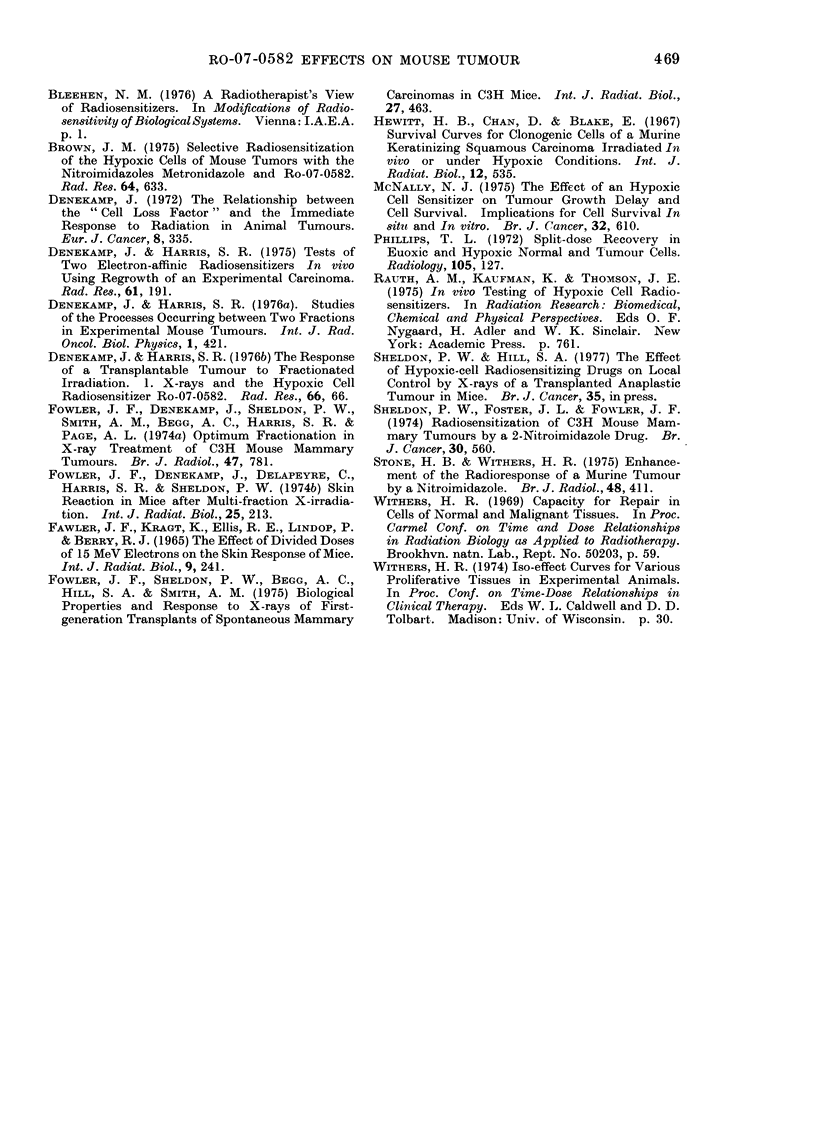# Radiosensitizing and cytocidal effects on hypoxic cells of RO-07-0582, and repair of x-ray injury, in an experimental mouse tumour.

**DOI:** 10.1038/bjc.1977.69

**Published:** 1977-04

**Authors:** S. A. Hill, J. F. Fowler

## Abstract

The delay in regrowth to 10 mm diameter of a transplanted carcinoma in mice was used to estimate the effect of the hypoxic-cell radiosensitizer Ro-07-0582. When 1 mg/g body wt. was given before a single dose of X-rays, a dose-enhancement ratio of 2-0 was found. When the drug was given immediately after irradiation, a large cytotoxic effect was observed, equivalent to an enhancement ratio of 1-3. These results were confirmed by determining the X-ray doses required for the local control of 50% of the tumours at 80 days after irradiation. The capacity of the tumour for repair of sublethal X-ray injury within 24 h was similar to that for several normal tissues.


Br. J. Cancer (1977) 35, 461

### RADIOSENSITIZING AND CYTOCIDAL EFFECTS ON HYPOXIC CELLS OF RO-07-0582, AND REPAIR OF X-RAY INJURY, IN AN EXPERIMENTAL MOUSE TUMOUR

S. A. HILL AND J. F. FOWLER

*From the Gray Laboratory of the Cancer Research Campaign, Mount Vernon Hospital, Northwood, Middlesex HA6 2RN*

Received 1 November 1976 Accepted 2 December 1976

**Summary.—The delay in regrowth to 10 mm diameter of a transplanted carcinoma in mice was used to estimate the effect of the hypoxic-cell radiosensitizer Ro-07-0582. When 1 mg/g body wt. was given before a single dose of X-rays, a dose-enhancement ratio of 2·0 was found. When the drug was given immediately after irradiation, a large cytotoxic effect was observed, equivalent to an enhancement ratio of 1·3. These results were confirmed by determining the X-ray doses required for the local control of 50% of the tumours at 80 days after irradiation.**

**The capacity of the tumour for repair of sublethal X-ray injury within 24 h was similar to that for several normal tissues.**

THE radiobiological properties of a squamous cell carcinoma which had previously been investigated by the excision and cell dilution method (Hewitt, Chan and Blake, 1967) were here studied, using the *in situ* methods of tumour regrowth and local control.

A major reason for this study was the exceptional finding of no radiosensitization in this tumour by the electron-affinic compound Ro-07-0582 using the dilution method (Hewitt, private communication). All other tumours so far studied have shown X-ray dose-enhancement ratios of about 2·0, using 1 mg/g body wt. of the drug. The question asked was whether this lack of radiosensitization was due to a difference in tumour type, or could be explained by the different methods of assessing tumour response.

In addition, information was sought about the shrinkage after irradiation, the proportion of hypoxic cells, and the amount of intracellular repair of sublethal injury that could occur in 24 h in this tumour.

### MATERIALS AND METHODS

The tumour, Squamous Carcinoma D, was obtained from Dr H. B. Hewitt. It arose spontaneously in his colony of WHT/Ht mice at the Gray Laboratory in 1965 as a keratinizing squamous carcinoma, but has since lost the ability to produce keratin and is now anaplastic. The origin of the tumour and its use in radiobiological cell survival studies has previously been described (Hewitt *et al.*, 1967).

For the present experiments, the tumours were implanted s.c. by trocar, on the ventral wall of the thorax of 8–12-week-old male WHT/Ht mice, under Penthrane anaesthesia. The tumours grew readily, forming fairly round, freely movable tumours, which were selected for irradiation when they reached a mean diameter of between 5·0 and 6·5 mm, at which time they had a volume-doubling time of 2 days.

The mice were anaesthetized with 60 mg/kg Na pentobarbitone and irradiated breathing air at room temperature. The X-irradiations were performed at 240 kV and 15 mA, using $\frac{1}{4}$ mm Cu and 1 mm Al filter to give a half-value layer of 1·3 mm Cu. The dose rate was 2·4/Gy min. The irradiations were performed as described by

Fowler *et al.* (1975), except that the mice were breathing air at room temperature (about 25°C) instead of warmed $O_2$. Four mice were irradiated simultaneously and turned through 180° halfway through each irradiation. To aid resuscitation, each mouse was given 0·5 mg bemigride i.p. after irradiation.

The following treatments were employed:

(a) Single doses of X-rays.

(b) Single doses of X-rays plus 1 mg/g body wt. of the radiosensitizing drug Ro-07-0582, dissolved in isotonic saline, injected i.p. 30 min before irradiation.

(c) Single doses of X-rays plus 1 mg/g Ro-07-0582 i.p. immediately after irradiation.

(d) Single doses of X-rays to the tumours made hypoxic by clamping off the blood supply to the tumour for 10 min before and during irradiation.

(e) Single doses of X-rays followed immediately by hypoxia, the blood supply to the tumour being occluded by a clamp for the time irradiation lasted plus 10 min.

(f) 1 mg/g Ro-07-0582 i.p., 30 min before irradiation, plus application of clamp 10 min before and during single doses of X-rays.

(g) 2 equal fractions of X-rays, 24 h apart, to tumours clamped 10 min before each irradiation.

After treatment, the tumours were measured 3 times per week over 3 perpendicular diameters, using vernier calipers, until they reached a mean diameter of at least 12 mm, at which time the animals were killed. If the size of a tumour at irradiation was not exactly 5·5 mm mean diameter, its growth curve was moved vertically so as to standardize it to 5·5 mm at irradiation (Denekamp and Harris, 1975). The number of days taken to reach 10 mm mean diameter (equivalent to 2·5 volume doublings) was then measured for each individual tumour, enabling a mean delay and standard error to be calculated for each dose group. This delay could then be plotted as " effect " *vs* X-ray dose.

Due to the high incidence of metastases, primarily to the lungs, long-term tumour-control experiments with this tumour were difficult, but 3 such experiments have been performed and the X-ray dose required for local control of 50% of the tumours (the $TCD_{50}$) was determined for single doses of X-rays alone, or with 1 mg/g Ro-07-0582 given 30 min before irradiation, or 1 mg/g Ro-07-0582 immediately after irradiation. Mice bearing this tumour also commonly displayed cachexia, becoming sick and losing weight rapidly as the tumour increased in size. To try to overcome this, the tumour was implanted into $F_1$ hybrid mice (WHT ♀ × CBA ♂). However, the delay in regrowth response of the tumour to X-rays was found to be different in these mice and their use was discontinued.

### RESULTS

A series of growth curves for tumours given a single dose of X-rays is shown in Fig. 1. The vertical bars represent

FIG. 1.—Growth curves for control and irradiated tumours (Squamous Carcinoma D) after single doses of X-rays. The vertical error bars are standard errors of the mean for each group of tumours on a particular day, and the numbers in brackets denote the number of tumours in each dose group.

the standard error of the mean diameter for each group of tumours on a particular day. A control curve is shown for animals which received no radiation. At all doses below 25 Gy, the tumours continued to grow for 1 or 2 days after irradiation, followed by shrinkage and eventual regrowth at different times.

Fig. 2 shows two dose-response curves constructed by estimating the time taken for each individual tumour to grow from 5·5 to 10 mm mean diameter and plotting the mean for the group of animals. It can be seen that the tumours in $F_1$ mice grew slightly faster. They also appeared to be less sensitive to X-rays than when implanted in the WHT mice (Fig. 2). However, if the delay induced by X-rays was divided by the normal doubling time, there would be no difference. All further experiments were performed using the WHT/Ht mice.

Two curves are shown in Fig. 3 for tumours irradiated in air or under hypoxic conditions, *i.e.*, 10 min after occluding the blood supply with a metal clamp. Also shown are two single points for animals whose tumours were made hypoxic immediately after irradiation, clamping being maintained for the same periods. The curve for control animals irradiated in air appears to be biphasic, with a break point at about 10 Gy, from which point it approaches the smooth and less steep hypoxic curve. The two " clamped

FIG. 2.—Dose-response curves plotted as the time taken to regrow to 10 mm mean diameter as a function of irradiation dose in gray. The two curves show the difference in response to single doses of X-rays, when the tumour is implanted into either WHT/Ht or F1 hybrid mice.

FIG. 3.—Dose-response curves for tumours in air-breathing animals irradiated with single doses of X-rays with or without clamping for 10 min before and during irradiation to make the tumours hypoxic. Two points are also shown for tumours which were clamped immediately after irradiation for the time of the X-ray dose plus 10 min; these fall on the " air " curve.

FIG. 4.—(a) Dose-response curves for tumours in air-breathing animals irradiated with single doses of X-rays with or without an i.p. injection of 1 mg/g Ro-07-0582 30 min before irradiation. The enhancement ratios for different levels of damage are given on the right hand side. Three points are also shown for Ro-07-0582 given 20 min before clamping the tumours, which were then irradiated 10 min later. (b) Dose-response curve for animals injected with 1 mg/g Ro-07-0582 immediately after irradiation, plotted against the " Air (control )" curve and the " Air 0582 Before " curve from Fig. 4a (dotted).

after X-rays " points fall exactly on the air curve, and it can therefore be said that in this tumour clamping alone has no effect.

Fig. 4 shows the data for animals irradiated in air with or without the drug Ro-07-0582 (1 mg/g). The control curve for X-rays only is the same biphasic curve as was plotted in Fig. 3. The curve for animals injected with 1 mg/g Ro-07-0582 30 min before irradiation is smooth, and indicates that there is an increasing sensitization with increasing dose of X-rays. The enhancement ratios range from 1·3, below the breakpoint on the X-ray-only curve, to over 2·0 at 10 Gy on the Ro-07-0582 curve. When Ro-07-0582 was given immediately after the X-rays (Fig. 4b) the points fell on a biphasic curve approximately parallel to the control line. This displacement from the air curve is interpreted as reflecting direct cytotoxic action of the drug, which is known to be specific for hypoxic cells, as discussed below. This cytotoxic action was equivalent to a post-irradiation enhancement ratio of between 1·2 and 1·4 and is consistent with killing about 50% of the hypoxic cells in the tumour.

Three points are shown in Fig. 4a for Ro-07-0582 given before clamping and then irradiating the tumours. They were not significantly different from the curve drawn through the corresponding points without clamping.

Corresponding data for the tumour control experiments are plotted in Fig. 5. Tumours of more than 4 mm mean diameter at 80 days were classified as local recurrences, those between 2 and 4 mm as ambiguous, and less than 2 mm diameter as locally controlled. In practice, none of the mice which survived to 80 days had palpable tumours, and none developed in the period between 80 and 150 days. However, by 80 days a significant proportion of the mice were lost due to metastases: almost 30% of mice in the X-ray-only experiment and 45% of mice receiving 1 mg/g Ro-07-0582. Only those animals which survived to 80 days, or were killed before this time with a recurrent tumour, were included in the analysis.

FIG. 5.—The proportion of tumours locally controlled at 80 days after irradiation plotted against X-ray dose. The three curves shown are (from right to left) for X-rays only, for X-rays immediately followed by i.p. injection of 1 mg/g Ro-07-0582 (ER 1·24 ± 0·12), and for i.p. injection of 1 mg/g Ro-07-0582 30 min before a single dose of X-rays (ER 2·0 ± 0·2). The horizontal bars represent standard errors of the mean at the $TCD_{50}$ level.

The $TCD_{50}$ and s.e. mean value were calculated using the computer programme devised by Dr E. H. Porter of the Glasgow Institute of Radiotherapeutics and Dr L. J. Peters of the Gray Laboratory, as described by Fowler *et al.* (1974*a*).

In Fig. 5 it can be seen that the $TCD_{50}$ was reduced from 46 to 23 Gy (*i.e.* ER = 2·0) when Ro-07-0582 was given before irradiation; and to 37 Gy (ER = 1·24) when the drug was given immediately afterwards.

Fig. 6 shows the regrowth of tumours made hypoxic by clamping, to single doses and to two equal fractions of X-rays 24 h apart. The extra dose needed ($D_2 - D_1$) to give a delay of 10 to 30 days when the irradiation was given in two fractions ($D_2$) rather than as a single dose ($D_1$) varied from 6 to 14 Gy. This represents the repair occurring within 24 h.

In order to make comparison with values obtained for well oxygenated normal tissues, all the hypoxic radiation doses should be divided by an assumed oxygen enhancement ratio (OER) which may be 2·5 to 3·0, including the hypoxic $D_2 - D_1$ value. In previous experiments on skin reactions, where reoxygenation was not a problem, it was found that the clamped-off $D_2 - D_1$ was indeed 2·5 to 3·0 times larger than the measured oxic $D_2 - D_1$ (Fowler *et al.*, 1965). In the present case we do not wish to underestimate the magnitude of $D_2 - D_1$, so the lower OER of 2·5 is assumed. The resulting ‘ oxic equivalent ” values of $D_2 - D_1$ have been plotted against the size of the first dose in Fig. 6b, together with data for normal tissues and 3 other tumours (see figure legend). It can be seen that the amount of repair in this tumour is similar to that in skin and intestine, and appears to be higher than in the other 3 tumours.

### DISCUSSION

### *General properties of the tumour*

This tumour does not shrink until at least 3 days after doses of 15 or 20 Gy, as shown in Fig. 1. Therefore it behaves differently from the carcinomas studied by Denekamp (1972) all except one of which shrank within a day after such doses. It does however shrink within 3 days after doses of 25 to 40 Gy.

Fig. 2 indicates that the transplanted tumours grew slightly more rapidly in

FIG. 6.—(a) Dose-reponse curves for clamped tumours given single doses or two equal fractions of X-rays separated by 24 h. (b) The capacity for "oxic" repair of sublethal injury in 24 h (*i.e.* $D_2 - D_1$) *vs* the size of the first of the two doses. Both axes record the measured hypoxic doses, divided by an assumed OER of 2·5. The dotted lines show results for some normal tissues. ✱ C3H mouse tumours (Fowler *et al.*, 1975), ◆ Carcinoma NT (24 h) and ◇ Sarcoma B (24 h; Denekamp and Harris, 1976*a*). The full line shows another set of results for Carcinoma NT at 48 h (Denekamp and Harris, 1976*b*). The tumour results were obtained by dividing the clamped-off $D_2 - D_1$ by an assumed OER of 2·5.

$F_1$ hybrids than in the WHT mice (*i.e.* at zero dose) and also that they were less radiosensitive in the $F_1$ mice.

*Proportion of hypoxic cells*

The proportion of hypoxic cells in the tumour can be estimated from the shape of the curve for aerobic tumours and its position relative to the clamped hypoxic curve (Fig. 3). The "in air" curve is biphasic, with an initial sensitive region up to 10 Gy, then becoming more resistant and "breaking" towards the clamped curve, as the hypoxic cells become important. The fraction of hypoxic cells can be estimated from the data in three ways:

(1) From the position of the breakpoint in the "in air" response curve which occurs at 10 Gy or possibly slightly lower. By assuming a $D_0$ value of 3·5 Gy for hypoxic cells, an oxygen enhancement ratio (OER) of between 2·5 and 3·0 and a $D_Q$ value of 4 to 6 Gy, the proportion of hypoxic cells can be computed to be between 10 and 30%. This value is not inconsistent with the estimate of 18% found by Hewitt *et al.* (1967) using excised tumours and a cell-dilution assay.

(2) From the displacement of the clamped-off with respect to the "in air" curve: they were not significantly different at high doses. By assuming the same survival-curve parameters, the hypoxic proportion can be calculated to be in the range 50 to 100%. However, this convergence of the curves at high doses may be due to a greater capacity for recovery of potentially lethal damage in chronically hypoxic cells than in those made acutely hypoxic by clamping, as suggested by McNally (private communication). This would lead to a higher estimate of the hypoxic fraction than obtained by a method in which the tumour was excised soon after irradiation.

(3) From converting the regrowth delay curves to "pseudo-survival curves" (Denekamp and Harris, 1975) by expressing the delay in terms of number of cell doublings needed to restore the tumour to its original size. This can be done by taking the time to regrow to 10 mm for the irradiated tumours, subtracting the corresponding time for the unirradiated (control) tumours and dividing by the volume-doubling time, *e.g.* 2 days. It was established that the time to grow from 8 to 10 mm was not significantly greater after doses of 5 to

25 Gy (2·75 ± 0·1), than in the unirradiated control group (2·63 ± 0·2). From the curves so drawn, the resistant, presumably hypoxic, portion of the aerobic curve can be extrapolated back to zero dose and gives an estimate of 10% hypoxic cells. Thus the proportion of hypoxic cells lies between 10% and 100%, depending upon the method of estimation used.

*Radiosensitization with Ro-07-0582*

Fig. 4a shows that, when 1 mg/g of the electron-affinic radiosensitizer is given before irradiation, a large dose-reduction factor or "sensitizer enhancement ratio" is obtained, between 2·0 and 2·4 over the dose range 18 to 25 Gy on the X-rays-only curve in air. This value is similar to other large values reported for several types of experimental tumour in mice using a variety of assay systems (Sheldon, Foster and Fowler, 1974; Denekamp and Harris, 1975; Brown, 1975; Begg, 1977; Sheldon and Hill, 1977; McNally, 1975; Stone and Withers, 1975; Rauth, Kaufman and Thomson, 1975; Bleehen, 1976; Denekamp and Stewart, personal communication). It differs however from the value of 1·0 reported by Hewitt for a cell-dilution experiment on this tumour, Squamous Carcinoma D, where two i.v. injections of 0·44 mg/g body wt. were given, 90 and 20 min before exposure to a dose of 18 Gy (private communication). A part of this difference can probably be explained by the direct cytotoxic effect of Ro-07-0582 on hypoxic cells.

The magnitude of this cytotoxic effect is indicated by the distance apart of the "Air (control)" curve and the "Air with 0582 after" curve in Fig. 4b. The enhancement ratio is not significantly different from 1·3, both above and below the breakpoint in the Air (control) curve. At doses below 10 Gy this cytotoxic effect could be the reason for the whole of the enhancement, corresponding to 2 Gy or 2–3 days of delay at doses from zero to about 8 Gy. At the dose of 18 Gy in air, as used by Dr Hewitt, the total sensitization ratio *in situ* was 2·0, of which 1·4 would of course not be seen in his method of excising tumours immediately after irradiation. The remaining difference, a factor of 2·0/1·4 = 1·4, might just be covered by the experimental errors in both methods.

The total effect on the tumour is of course what is measured *in situ*: both effects contribute usefully to the eradication of hypoxic cells. The cytotoxic effect is large in the present tumours. Post-radiation (*i.e.* cytotoxic) enhancements of 1·17 (Brown, personal communication), 1·1–1·4 (Denekamp and Harris, 1975), and 1·0–1·2 (Begg, 1977), have been reported, but in two other types of mouse carcinoma no significant post-irradiation enhancement was observed (Sheldon *et al.*, 1974; Sheldon and Hill, 1977).

To test whether the drug was in fact penetrating to all the naturally hypoxic cells, the tumours were made anoxic by clamping, after an injection of Ro-07-0582 but before irradiating. This should demonstrate the maximum sensitizing effect of the drug, since all the cells have been made totally hypoxic. The results at the two X-ray doses tested (Fig. 4a) were the same as if the tumours had not been clamped, thereby indicating that the drug is indeed reaching the hypoxic cells.

*Repair in 24 h*

Fig. 6 shows the response curves for single doses and equal doses given 24 h apart for tumours clamped off to make all the cells hypoxic. In those circumstances, the value of $D_2 - D_1$ represents repair of the cells which are made uniformly hypoxic during irradiation, without the artefact of re-oxygenation. The values of $D_2 - D_1$ from Fig. 6a increased from about 13 Gy to 15 Gy as $D_2$ was varied from 30 to 40 Gy. If $D_2 - D_1$ is divided by an arbitrarily assumed oxygen enhancement ratio of 2·5, the resulting "repair of oxic cells" estimated is

5·2–6·0 Gy. The extent of this repair will vary with the dose per fraction ($\frac{1}{2}D_2$) as shown in Fig. 6b. In the same figure the oxic repair in 24 h measured in skin (Fowler *et al.*, 1974*b*) and in the intestinal mucosa of the jejunum in 3 h (Withers, 1969) and of the colon in 24 h (Withers, 1974) is shown plotted against the size of the first of the 2 radiation doses used. The repair measured in 3 other types of tumour in our laboratory is also shown. In contrast to the present tumour, they were not anaplastic. All the tumour results were obtained by dividing the repair measured in clamped-off tumours by the OER of 2·5.

It can be seen from Fig. 6b that, although the other 3 tumour results are lower than the normal tissue results, this is not so for the present tumour. Repair appears to be similar to that in some of the normal tissues. If an OER of 3 had been assumed instead of 2·5, the estimated repair in the tumour would be correspondingly lower, but the general conclusion would not be altered. In this tumour there is no evidence of less repair in 24 h than in normal tissues, as indeed Withers (1969) concluded. Nevertheless, Phillips (1972) considered that the recovery of sarcomas and mammary carcinomas was significant but was " less than that of the normal skin and gut ". It seems clear that different types of tumour demonstrate different amounts of repair in 24 h, large amounts perhaps being associated with appreciable repair of potentially lethal injury.

### CONCLUSIONS

1. This squamous carcinoma did not shrink until more than 3 days after 15–20 Gy.

2. Three different estimates of the proportion of hypoxic cells in the tumour gave values ranging from 10% to 100%. No conclusion can yet be made about the differences, although the higher value may be partly explained by more recovery of PLD occurring in chronically hypoxic cells than in acutely hypoxic (clamped) tumours, particularly after the higher doses of radiation used.

3. Large enhancement ratios of 2·0–2·4 were observed at X-ray doses above 9 Gy, when 1 mg/g of Ro-07-0582 was injected before irradiation. The value of 1·0 (no enhancement), found earlier by an excision method, may be explained at least partly by a large cytotoxic effect of the drug on hypoxic cells left *in situ*.

4. A large effect of cytotoxicity on hypoxic cells was observed by injecting Ro-07-0582 immediately after irradiation, corresponding to an enhancement ratio of about 1·3. In some other types of tumour this cytotoxicity cannot be demonstrated.

5. The results of tumour-control experiments agreed with those of the regrowth-delay experiments, for both true sensitization and post-irradiation enhancement due to hypoxic cell cytotoxicity.

6. The 24-h repair of sublethal X-ray injury in tumour cells, irradiated with the blood supply occluded to make them uniformly hypoxic, was similar to that for some normal tissues.

7. The shape of the dose-response curves in Fig. 3 is consistent with appreciable repair of potentially lethal damage in hypoxic cells specifically. This repair of potentially lethal damage may be the cause of the large amount of repair mentioned in the previous conclusion.

We should like to thank Dr H. B. Hewitt for providing the tumour; Angela Walder, Ann Marriott and Suzanne Bull for the breeding and care of the mice; Dr J. Denekamp for helpful discussions; Sandra Fairman for help with implanting the tumour; Roche Products Ltd., Welwyn Garden City, for supplying the Ro-07-0582; and the Cancer Research Campaign for support.

### REFERENCES

BEGG, A. C. (1977) The Use of $^{125}$I-iododeoxyuridine to Measure Hypoxic Cell Radiosensitization by Ro-07-0582 in a Solid Murine Tumour. *Rad. Res.* (in press).

BLEEHEN, N. M. (1976) A Radiotherapist's View of Radiosensitizers. In *Modifications of Radiosensitivity of Biological Systems.* Vienna: I.A.E.A. p. 1.

BROWN, J. M. (1975) Selective Radiosensitization of the Hypoxic Cells of Mouse Tumors with the Nitroimidazoles Metronidazole and Ro-07-0582. *Rad. Res.* **64**, 633.

DENEKAMP, J. (1972) The Relationship between the "Cell Loss Factor" and the Immediate Response to Radiation in Animal Tumours. *Eur. J. Cancer*, **8**, 335.

DENEKAMP, J. & HARRIS, S. R. (1975) Tests of Two Electron-affinic Radiosensitizers *In vivo* Using Regrowth of an Experimental Carcinoma. *Rad. Res.*, **61**, 191.

DENEKAMP, J. & HARRIS, S. R. (1976*a*). Studies of the Processes Occurring between Two Fractions in Experimental Mouse Tumours. *Int. J. Rad. Oncol. Biol. Physics*, **1**, 421.

DENEKAMP, J. & HARRIS, S. R. (1976*b*) The Response of a Transplantable Tumour to Fractionated Irradiation. 1. X-rays and the Hypoxic Cell Radiosensitizer Ro-07-0582. *Rad. Res.*, **66**, 66.

FOWLER, J. F., DENEKAMP, J., SHELDON, P. W., SMITH, A. M., BEGG, A. C., HARRIS, S. R. & PAGE, A. L. (1974*a*) Optimum Fractionation in X-ray Treatment of C3H Mouse Mammary Tumours. *Br. J. Radiol.*, **47**, 781.

FOWLER, J. F., DENEKAMP, J., DELAPEYRE, C., HARRIS, S. R. & SHELDON, P. W. (1974*b*) Skin Reaction in Mice after Multi-fraction X-irradiation. *Int. J. Radiat. Biol.*, **25**, 213.

FAWLER, J. F., KRAGT, K., Ellis, R. E., LINDOP, P. & BERRY, R. J. (1965) The Effect of Divided Doses of 15 MeV Electrons on the Skin Response of Mice. *Int. J. Radiat. Biol.*, **9**, 241.

FOWLER, J. F., SHELDON, P. W., BEGG, A. C., HILL, S. A. & SMITH, A. M. (1975) Biological Properties and Response to X-rays of First-generation Transplants of Spontaneous Mammary Carcinomas in C3H Mice. *Int. J. Radiat. Biol.*, **27**, 463.

HEWITT, H. B., CHAN, D. & BLAKE, E. (1967) Survival Curves for Clonogenic Cells of a Murine Keratinizing Squamous Carcinoma Irradiated *In vivo* or under Hypoxic Conditions. *Int. J. Radiat. Biol.*, **12**, 535.

McNALLY, N. J. (1975) The Effect of an Hypoxic Cell Sensitizer on Tumour Growth Delay and Cell Survival. Implications for Cell Survival *In situ* and *In vitro. Br. J. Cancer*, **32**, 610.

PHILLIPS, T. L. (1972) Split-dose Recovery in Euoxic and Hypoxic Normal and Tumour Cells. *Radiology*, **105**, 127.

RAUTH, A. M., KAUFMAN, K. & THOMSON, J. E. (1975) *In vivo* Testing of Hypoxic Cell Radiosensitizers. In *Radiation Research: Biomedical, Chemical and Physical Perspectives.* Eds O. F. Nygaard, H. Adler and W. K. Sinclair. New York: Academic Press. p. 761.

SHELDON, P. W. & HILL, S. A. (1977) The Effect of Hypoxic-cell Radiosensitizing Drugs on Local Control by X-rays of a Transplanted Anaplastic Tumour in Mice. *Br. J. Cancer*, **35**, in press.

SHELDON, P. W., FOSTER, J. L. & FOWLER, J. F. (1974) Radiosensitization of C3H Mouse Mammary Tumours by a 2-Nitroimidazole Drug. *Br. J. Cancer*, **30**, 560.

STONE, H. B. & WITHERS, H. R. (1975) Enhancement of the Radioresponse of a Murine Tumour by a Nitroimidazole. *Br. J. Radiol.*, **48**, 411.

WITHERS, H. R. (1969) Capacity for Repair in Cells of Normal and Malignant Tissues. In *Proc. Carmel Conf. on Time and Dose Relationships in Radiation Biology as Applied to Radiotherapy.* Brookhvn. natn. Lab., Rept. No. 50203, p. 59.

WITHERS, H. R. (1974) Iso-effect Curves for Various Proliferative Tissues in Experimental Animals. In *Proc. Conf. on Time-Dose Relationships in Clinical Therapy.* Eds W. L. Caldwell and D. D. Tolbart. Madison: Univ. of Wisconsin. p. 30.